# Association Between the Dutch Mediterranean‐Dietary Approaches to Stop Hypertension Intervention for Neurodegenerative Delay (MIND‐NL) Diet Adherence and Systemic Tryptophan Metabolites in Older Adults at Risk of Cognitive Decline: An Exploratory Study

**DOI:** 10.1002/mnfr.70377

**Published:** 2026-01-16

**Authors:** Sonja Beers, Lisette C. P. G. M. de Groot, Mark R. van Loenen, Lianne B. Remie, Mechteld Grootte Bromhaar, Andrea Anesi, Urska Vrhovsek, Joukje M. Oosterman, Esther Aarts, Mara P. H. van Trijp, Yannick Vermeiren

**Affiliations:** ^1^ Division of Human Nutrition and Health Wageningen University & Research Wageningen the Netherlands; ^2^ Radboud University Donders Institute for Brain, Cognition and Behaviour Nijmegen the Netherlands; ^3^ Unit of Metabolomics, Department of Food Quality and Nutrition Research and Innovation Centre, Fondazione Edmund Mach (FEM) San Michele all'Adige Italy

**Keywords:** dietary pattern, kynurenine pathway, latent change model, serotonin

## Abstract

Tryptophan (TRP) metabolism is emerging as a focus of investigation in Alzheimer's disease research. In addition, diet might impact TRP's metabolic fate. The aim of this study was to explore the association between adherence to the Dutch Mediterranean‐Dietary Approaches to Stop Hypertension (DASH) Intervention for Neurodegenerative Delay (MIND‐NL) diet and systemic TRP metabolite levels and their related ratios in older adults at risk of cognitive decline. Data of the HELI multidomain lifestyle intervention study (*n* = 82) were used. Dietary intake data (FFQ) and fasted plasma levels were collected at baseline and 26 weeks follow‐up. Bivariate Latent Change Score Models (LCSMs) were applied to assess both level‐level and change‐change associations, adjusted for lifestyle factors. Changes in MIND‐NL diet adherence were significantly inversely associated with changes in the kynurenine:large neutral amino acids ratio (path coefficient −0.457, SE:0.206, *p* = 0.03) and kynurenine:tryptophan ratio (path coefficient −0.558 SE:0.235, *p* = 0.02). In addition, MIND‐NL diet adherence was associated with lower levels of the neurotoxic metabolite quinolinic acid (path coefficient −0.186, SE:0.0700, *p* = 0.008), but within the crude model only. Our findings suggest that greater adherence to the MIND‐NL diet may contribute to decreased activation of the kynurenine pathway.

## Graphical abstract

Following the MIND diet may support brain health in older adults at risk of cognitive decline by influencing tryptophan (TRP) metabolism. Plasma samples and dietary intake data from the HELI intervention trial were used. Bivariate latent change score modeling analysis showed that greater adherence to the MIND diet was linked to higher tryptophan levels, lower levels of quinolinic acid, a harmful neurotoxic metabolite, and lower levels of the kynurenine/TRP ratio. This suggests the diet reduces activation of the kynurenine pathway, involved in neurodegeneration.

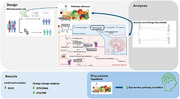

**Abbreviations**
5‐HIAA: 5‐hydroxyindole‐3‐acetic acid
5‐HT: serotonin
AD: Alzheimer's disease
BBB: blood–brain barrier
FFQ: Food Frequency Questionnaire
KA: kynurenic acid
KYN: Kynurenine
LNAAs: large neutral amino acids
MIND: Mediterranean‐DASH Intervention for Neurodegenerative Delay
PA: Picolinic acid
PSQI: Pittsburgh Sleep Quality Index
PSS: Perceived Stress Scale
QA: quinolinic acid
SQUASH: Short Questionnaire to Assess Health‐enhancing physical activity
TRP: Tryptophan
XA: Xanthurenic acid

## 1 Introduction

Alzheimer's disease (AD) is a progressive neurodegenerative disorder marked by cognitive decline, behavioral impairment, and, eventually, the loss of functional abilities. AD is best conceptualized as a continuum due to its progressive nature, beginning with asymptomatic, preclinical changes that advance to mild cognitive impairment (MCI) and, ultimately, to clinically evident dementia [1]. Despite extensive research into AD, effective disease‐modifying interventions to slow down or halt pathology are still limited [2], which stresses the importance of research into prevention [3, 4]. Diet has emerged as one of the modifiable factors in the prevention of dementia. However, the physiological mechanisms by which diet may influence the pathological processes involved in the AD continuum remain insufficiently understood, making it challenging to comprehensively understand the role of diet in dementia prevention [5].

Tryptophan (TRP) metabolism is receiving increased scientific interest within the field of AD pathophysiology. Tryptophan is an essential amino acid that cannot be synthesized by the body and should be obtained through diet. Tryptophan is catabolized in the body via multiple pathways: approximately 90% is catabolized through the kynurenine pathway, which leads to the production of metabolites involved in immune regulation and neuroactive compounds, while a mere ∼5% is metabolized via the hydroxylation pathway to produce the neurotransmitters serotonin (5‐hydroxytryptamine; 5‐HT) and melatonin [6]. A balanced metabolism between neuroprotective and neurotoxic metabolites within these pathways is crucial, as shifts toward neurotoxic metabolites, such as quinolinic acid (QA), in the kynurenine pathway, might induce neuronal damage [6]. Changes in TRP metabolism have been observed in AD patients [7]. For instance, higher plasma levels of QA have been found in AD patients compared to non‐AD controls [8]. Furthermore, shifts in TRP metabolism were shown to affect hallmarks of AD [9], including inflammation [10], oxidative stress [10], glucose metabolism [11], and protein aggregation [12], evidenced in both human as well as animal studies. Therefore, TRP catabolism might be a promising target to slow down cognitive decline and delay dementia onset.

To assess and represent metabolic dynamics of TRP metabolism, several biomarker ratios have recently been introduced. Transport across the blood–brain barrier (BBB) is determined not only by peripheral TRP and kynurenine (KYN) levels but also by the presence of competing large neutral amino acids (LNAAs), including leucine, isoleucine, tyrosine, phenylalanine, and valine [13]. Therefore, the ratios of TRP/LNAA and KYN/LNAA provide a relative estimation of the transport of TRP and KYN across the BBB. In addition, the ratio of KYN/TRP is an indicator for the flux of TRP to be used in the kynurenine pathway, and is a marker of the enzymatic conversion rate. The 5‐HT/TRP ratio represents a marker of serotonin synthesis from TRP. Furthermore, the kynurenic acid (KA)/QA ratio is an indicator for relative neuroprotection [14].

Diet might impact TRP metabolism through several mechanisms. One route is via dietary antioxidants, which can reduce chronic inflammation and thereby inhibit the activation of indoleamine 2,3‐dioxygenase (IDO), the rate‐limiting enzyme of the kynurenine pathway [14, 15, 16, 17]. IDO is upregulated in response to inflammation. Reducing its activity might reduce the production of neurotoxic metabolites of the kynurenine pathway and increase TRP's availability for 5‐HT synthesis [14]. Antioxidants may also protect tetrahydrobiopterin (BH4), a cofactor that is essential for 5‐HT synthesis. In addition, vitamin B6 serves as a cofactor in the enzymatic conversion of KYN into KA, one of the acclaimed neuroprotective metabolites. Trace elements such as Mn^2^^+^, Zn^2^^+^, Co^2^^+^, Cu^2^^+^, and Fe^3^^+^ have also been indicated to regulate the enzymatic activity of the kynurenine pathway [15].

Given the complex neurochemical interactions of TRP metabolites with dietary intake, a balanced diet seems essential for maintaining an optimal bioavailability to the brain. Although one would expect the individual nutrients from diet to have profound effects on TRP metabolism, there are only a handful of human studies investigating the associations of whole diets [16, 17, 18, 19]. The Mediterranean‐Dietary Approaches to Stop Hypertension (DASH) Intervention for Neurodegenerative Delay (MIND) diet, a dietary pattern combining elements of both the Mediterranean and DASH diets, has been associated with a lower incidence of AD and cognitive decline in observational studies [20]. In addition, untargeted metabolomic studies recently suggested an association between adapted MIND diet adherence scores and TRP metabolism in generally healthy middle‐aged and older adults [18, 19]. However, this specific pathway should be more thoroughly investigated in close relation to the MIND diet. In particular, there is a lack of research on how changes in MIND diet adherence are associated with alterations in TRP metabolites and its ratios.

To assess the MIND diet in relation to TRP metabolism, selecting an appropriate study population might be crucial. Given our focus on the AD continuum, particularly its earliest preclinical stage, it might not be the most suitable approach to include an overall healthy older population. Instead, this study includes older adults with modifiable lifestyle‐related (cardiovascular) risk factors, aiming to capture a population more likely to be in the asymptotic, preclinical stage of AD. Since no biofluid or neuroimaging biomarkers exist to definitively identify this stage, targeting at‐risk individuals increases the likelihood of studying relevant metabolic changes.

Therefore, the aim of this study is to explore both cross‐sectional as well as change‐change associations between adherence to the MIND diet and systemic TRP metabolite levels in older adults at increased risk of cognitive decline. Specifically, we aim to assess TRP metabolite ratios as potential markers of metabolic balance between neuroprotective and neurotoxic pathways.

## 2 Experimental Section

### 2.1 Study Design

Data from the HELI study were used for analyses. The HELI study is a 26‐week multicenter randomized multidomain lifestyle intervention trial in older adults at risk of cognitive decline, focused on investigating brain health [21]. The study protocol was approved by the Medical Research Ethics Committee (MREC) Oost‐Nederland (ToetsingOnline filenumber NL78263.091.21, ClinicalTrials.gov ID NCT05777863). Each participant provided written consent.

In short, the multidomain lifestyle intervention comprised five domains: diet (MIND‐NL), physical activity, sleep, stress management/mindfulness, and cognitive training. The intervention group received a weekly, structured and group‐based, high‐intensity intervention program. The control group received a low‐intensity program, by sending general lifestyle‐related health information once every 2 weeks, without any further leads on how to improve or adjust their lifestyle. More details about the intervention can be found in the article by van Loenen et al. [21]. The primary outcome of this study is the changes in brain activity, cerebral perfusion levels, and peripheral immunometabolic markers after the 26‐week intervention [21]. The current study includes secondary outcome measures.

Within our analyses, both baseline and follow‐up data were used. Intervention and control groups were combined for analysis, as the focus was on actual dietary changes over time, regardless of group allocation. Both groups comprised individuals who improved or declined in MIND‐NL diet adherence, allowing for meaningful modeling of within‐person change.

### 2.2 Participants

Participants were recruited between May 2022 and October 2023. Individuals had to be (1) between 60 and 75 years old at the moment of signing written informed consent, (2) fluent in Dutch, and (3) score ≥2 points on the self‐reported “Modifiable cardiovascular risk factor scale” (Table S1). The risk factor scale indicates increased risk for cognitive decline based on risk factors that are modifiable by lifestyle changes (adapted from CAIDE [22]). Individuals were excluded when they (1) concurrently participated in other intervention trials, (2) were technological illiterate, (3) had no internet access from home, (4) were cognitively impaired as determined by Telephone Interview for Cognitive Status (TICS‐M1) score of <23 points, (5) had a clinical diagnosis of cerebrovascular event, neurological disease (including MCI and dementia), current malignant disease, psychiatric disorder, moderate to severe cardiovascular disease, revascularization surgery in the last 12 months, inflammatory bowel disease, visual impairment (which is uncorrectable with visual aids), and hearing or communicative impairment (which is uncorrectable with hearing aids), or (5) had MRI contradictions.

A total of 102 participants were included in the intervention. For the current study, participants were excluded in case of missing blood samples and diet scores.

### 2.3 Diet Assessment

Participants completed a short validated FFQ (MIND‐NL‐Eetscore‐FFQ) at baseline and at 26 weeks follow‐up [23]. The short FFQ includes 72 food items and 41 questions and assesses both adherence to the 2015 Dutch dietary guidelines and the MIND‐NL diet. The short FFQ has been validated and showed acceptable ranking properties. The questions are organized chronologically, beginning with breakfast and progressing through to late evening. The reference period was the previous month. Intakes were estimated using standard portion sizes and commonly used household measures. Average daily intake, in grams, of food items was calculated by multiplying the frequency of consumption by the portion size. The MIND‐NL‐Eetscore‐FFQ was administered using a web‐based assessment tool (www.eetscore.nl).

The dietary adherence score, that is, the MIND‐NL score, reflects dietary intake of 15 food components. Nine of the 15 food groups are recommended: green leafy vegetables, other vegetables, berries and strawberries, whole grains, nuts, fish, poultry, beans and legumes, and olive oil. Six of the 15 food groups are advised to limit: full‐fat cheese, butter and sticky margarines, red and processed meat, take‐out meals, fried foods and snacks, and cookies, pastries, and sweets. Each food component can be scored 0, 0.5, or 1.0. The MIND‐NL score summarized the individual food component scores to a score between 0 and 15. A higher score reflects better adherence to the MIND‐NL diet (Table S2).

### 2.4 Plasma Tryptophan Metabolites and Large Neutral Amino Acid (LNAA) Analyses

Participants consumed a standardized meal on the evening prior to blood drawing. Fasting whole blood was collected in EDTA tubes during the morning at baseline and 26 weeks, followed by centrifugation for 10 min at 1200 × *g* at 4°C, resulting in fasting plasma aliquots. Plasma aliquots were stored at −80°C until analyses. Plasma samples from both baseline and 26‐weeks were analyzed in the same batch. For the analyses, ultra high‐performance liquid chromatography‐electrospray ionization‐tandem mass spectrometry (UHPLC‐ESI‐MS/MS) was applied with an AB Sciex 6500+ triple quadrupole coupled to a Shimadzu LC‐30 AD pump (AB Sciex, Milan, Italy). The metabolites were analyzed according to a previously described method [24].

The following TRP metabolites of the kynurenine and hydroxylation pathways were measured: TRP, KYN, KA, xanthurenic acid (XA), QA, picolinic acid (PA), 5‐HT, and 5‐hydroxyindole‐3‐acetic acid (5‐HIAA). The LNAAs measured were leucine, isoleucine, tyrosine, phenylalanine, and valine. Additionally, the method also allowed for quantification of microbiota‐derived tryptophan metabolites (Table S4).

LNAAs have been interpreted and calculated as the sum of leucine, isoleucine, tyrosine, phenylalanine, and valine concentration, excluding TRP. The following ratios are calculated and used in the analyses: TRP/LNAA (multiplied by 10), KYN/LNAA (multiplied by 1000), KYN/TRP ratio (multiplied by 100), 5‐HT/TRP ratio (multiplied by 100), and KA/QA ratio.

### 2.5 Other Measures

Anthropometric measurements, blood samples, and questionnaires about background (age, education level), lifestyle, and general health were collected at baseline and 26 weeks. Participants recorded physical activity with the Short Questionnaire to Assess Health‐enhancing physical activity (SQUASH) [25]. Total activity score (minutes of activity per week*intensity) was reported as a measure of physical activity. Sleep quality was measured with the Pittsburgh Sleep Quality Index (PSQI) questionnaire [26]. Stress perception was measured with the Perceived Stress Scale (PSS) questionnaire [27]. Although cognitive activities were also part of the intervention, adherence to this domain was not determined.

Via a finger prick, inflammation markers were measured. The inflammation marker C‐reactive protein (CRP) was measured with the Quick Read Go (Aidian Oy, Finland). For the analysis of the white blood cell (WBC) count, the HemoCue WBC DIFF micro cuvette (HemoCue AB, Ängelhom, Sweden) was directly placed in the HemoCue WBC DIFF (HemoCue AB, Ängelhom, Sweden) and analyzed. WBC was measured by analyzing the neutrophils, lymphocytes, monocytes, eosinophils, and basophils. Finally, plasma AD biomarker levels were measured (amyloid‐beta 1‐42/1‐40, neurofilament light chain and glial fibrillary acidic protein) applying Simoa as described in [28].

### 2.6 Statistical Analyses

Normality of continuous variables was assessed using histograms and Q–Q plots. Skewed metabolite distributions were log‐transformed prior to analysis.

**FIGURE 1:**
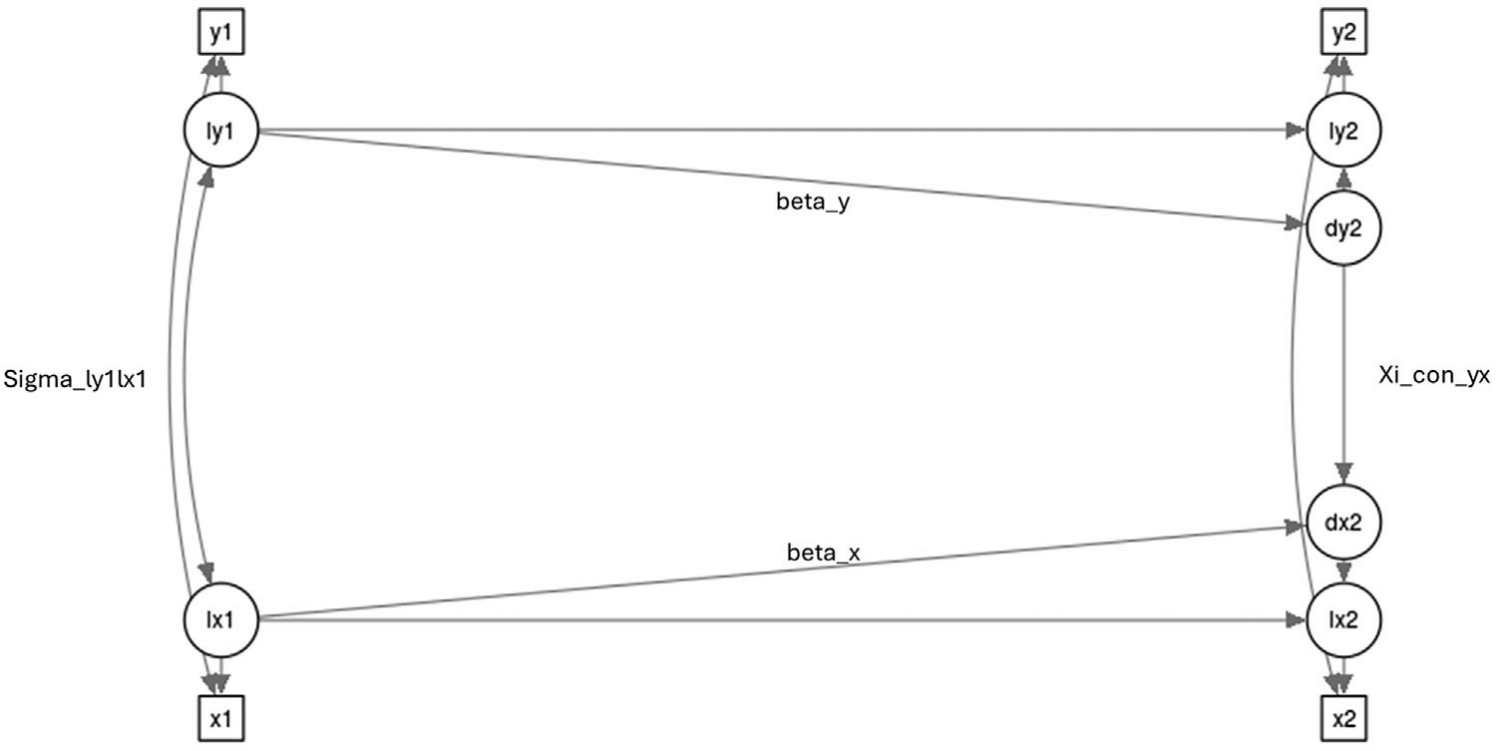
Path diagram of bivariate LCSM. Squares: Observed variables; circles: Latent variables; Single‐headed arrows: Regressions; Double‐headed arrows = Covariance. Sigma_ly1lx1 represents the cross‐sectional association of baseline MIND‐NL diet and baseline metabolite. Xi_con_yx represent the association between the change in MIND‐NL diet and the change in metabolite. Figure generated in shinychange.

To assess whether a change in MIND‐NL diet adherence was associated with a change in systemic tryptophan metabolite concentrations and related ratios, we applied bivariate Latent Change Score Models (LCSMs) within a structural equation modeling (SEM) framework. General level (latent intercept) and change (latent difference score) were simultaneously estimated, and their associations were studied. Specifically, the model estimated whether changes in MIND diet adherence were associated with concurrent changes in metabolite concentrations, controlling for baseline levels of both variables (Figure 1). In addition to baseline adjustment, the model incorporated residual change of BMI, physical activity, sleep, and perceived stress as covariates of the latent change scores to account for concurrent lifestyle changes. Corresponding baseline values of these covariates, along with age and sex, were included as predictors of the latent intercepts to account for potential confounding.

To address missing data at follow‐up, Full Information Maximum Likelihood estimation was used. Model fit was examined using Chi‐Square (*p* > 0.05), Comparative Fit Index (CFI, ≥0.95), Root Mean Square Error of Approximation (RMSEA, <0.06), and Standardized Root Mean Square Residual (SRMR, <0.08) (Tables S3 and S5) [29].

One extreme outlier in plasma KYNA levels at baseline (10.4 µM), representing a >100‐fold difference compared to follow‐up, was excluded due to implausibility.

All analyses were conducted using R (Version 4.4.1). LCSMs were estimated using the R package *lavaan* [30].

## 3 Results

### 3.1 Characteristics of the Study Participants

Of the 102 participants, blood measurements and dietary data were available for 82 participants at baseline and 76 participants at 26 weeks.

Among the 82 participants included at baseline, the mean age was 66.6 ± 4.3 years, 62.2% were female, and the mean BMI was 29.8 ± 5.4 kg/m^2^. The overall MIND diet score was 8.5 ± 2.1, the total physical activity score was 7194 ± 3902, participants had overall poor sleep quality (PSQI score: 5 [4.0, 7.8]), and overall low stress was perceived (PSS score: 12.1 ± 3.9) (Tables 1, 2).

**TABLE 1:** Baseline characteristics (*N* = 82).

Characteristic	Mean (SD)
Age	66.6 (4.3)
Sex (female), n (%)	51 (62.2)
Education level,^a^ n (%)	
Low	9 (11.0)
Medium	30 (36.6)
High	43 (52.4)
MoCA (/30)	27.1 (2.1)
Metabolic parameters	
Waist circumference (cm)	102.2 (14.5)
Waist‐hip ratio	0.95 (0.10)
Systolic blood pressure (mmHg)	149.1 (19.6)
Diastolic blood pressure (mmHg)	89.0 (12.3)
Hypertension,^b^ n (%)	57 (67.9)
Triglycerides (mmol/L), median [IQR]	1.47 [1.16, 2.20]
Total cholesterol (mmol/L)	5.60 (1.18)
HDL (mmol/L)	1.46 (0.43)
LDL (mmol/L)	3.41 (0.98)
Glucose (mmol/L), median [IQR]	5.80 [5.50, 6.40]
Inflammation markers	
CRP (mg/L), median [IQR]	1.5 [1.2, 4.0]
White Blood Cell count (x10^9^/L)	6.3 (1.9)
Neutrophil count (x10^9^/L)	3.5 (1.4)
Lymphocyte count (x10^9^/L)	2.4 (0.69)
Monocyte count (x10^9^/L), median [IQR]	0.3 [0.2, 0.3]
Eosinophil count (x10^9^/L), median [IQR]	0.1 [0.1, 0.2]

*Note*: Data are presented as mean ± SD, unless indicated otherwise.

^a^ Based on the International Standard Classification of Education (ISCED 2011) guidelines (low, medium, high education categorization).

^b^ Hypertension: Systolic blood pressure ≥140 and/or diastolic blood pressure ≥90 mmHg.

**TABLE 2:** Baseline and follow‐up characteristics related to diet, lifestyle, AD biomarkers, and tryptophan metabolite concentrations.

Characteristic	Baseline *N* = 82	Follow‐up *N* = 76	*p*‐value
MIND‐NL score (/15)	8.5 (2.1)	9.9 (2.1)	**<0.001**
			
BMI (kg/m^2^)	29.8 (5.4)	29.6 (5.6)	0.06
SQUASH, total physical activity score^a^	7194 (3902)	6353 (3784)	**0.02**
PSQI, global score, median [IQR]	5.0 [4.0, 7.8]	5.0 [3.0, 7.0]	0.15
PSS, score	12.1 (3.9)	10.4 (5.3)	**0.002**
Metabolite levels in plasma			
Tryptophan (µM), median [IQR]	79.6 [69.1, 90.3]	79.3 [72.4, 89.2]	0.81
Kynurenine (µM), median [IQR]	1.44 [1.24, 1.74]	1.55 [1.27, 1.82]	0.33
Kynurenic acid (nM),^e^ median [IQR]	55.8 [45.3, 72.6]	56.1 [46.4, 73.9]	0.91
Xanthurenic acid (nM), median [IQR]	449 [387, 517]	438 [386, 497]	0.39
Quinolinic acid (nM), median [IQR]	431 [331, 560]	441 [367, 563]	0.71
Picolinic acid (nM)	44.9 (17.0)	42.6 (18.9)	0.21
Serotonin (µM)	1.38 (0.74)	1.13 (0.68)	**0.005**
5‐hydroxyindole‐3‐acetic acid (nM), median [IQR]	133 [109, 157]	123 [98.0, 151]	0.74
Ratios			
TRP/LNAA^b^	1.06 (0.16)	1.07 (0.15)	0.21
KYN/LNAA^d^	1.99 (0.45)	2.06 (0.60)	0.08
KYN/TRP^c^	1.92 (0.49)	1.96 (0.65)	0.30
5‐HT/TRP^c^	1.74 (0.91)	1.45 (0.93)	**0.01**
KA/QA^b^, ^e^	1.40 (0.61)	1.45 (0.89)	0.70
AD‐related biomarkers			
NfL (pg/mL), (*n* = 82)	14.3 (5.9)	14.5 (6.0)	0.20
Aβ42/40 (ratio), median [IQR] (*n* = 82)	0.060 [0.050, 0.066]	0.059 [0.048, 0.068]	0.36
GFAP (pg/mL) (*n* = 82)	74.5 (23.4)	77.3 (26.1)	**0.008**

*Notes*: Data are presented as mean ± SD, unless indicated otherwise. Significant p values are presented in bold. Normal deviated variables tested with paired *t*‐test and non‐normal distributed values with Wilcoxon Signed‐Rank Test. PSQI questionnaire: A higher score represents worse sleep quality, possible range between 0 and 21. A score of ≥5 points indicate poor sleep quality [26]; PSS questionnaire: possible range between 0 and 40, score of 0–13: low stress, 14–26 moderate stress, 17–40 high perceived stress [27].

^a^ Baseline: *N* = 80, Follow‐up: *N* = 72.

^b^ Multiplied by 10.

^c^ Multiplied by 100.

^d^ Multiplied by 1000.

^e^ Baseline *N* = 79, follow‐up: *N* = 74.

Abbreviations: Aβ, amyloid‐beta; GFAP, glial fibrillary acidic protein; KA, kynurenic acid; KYN, kynurenine; LNAA, large neutral amino acids; MIND, Mediterranean‐DASH Intervention for Neurodegenerative Delay; NfL, neurofilament light chain; PSQI, Pittsburgh Sleep Quality Index; PSS, Perceived Stress Scale; QA, quinolinic acid; SQUASH, Short Questionnaire to Assess Health‐enhancing Physical Activity; TRP, tryptophan; 5‐HT, serotonin.

Overall, there was a significant increase in MIND‐NL diet score between baseline and follow‐up, with a mean difference of 1.4 (95% CI: 0.9, 1.9) (Table 2). Twenty‐eight (36.8%) participants improved their dietary adherence score by at least 2 points, and four (5.3%) participants decreased their dietary adherence score by at least 2 points.

### 3.2 Systemic TRP Metabolites of the Kynurenine and Hydroxylation Pathways

Associations between MIND‐NL diet and tryptophan metabolite concentrations are summarized in Table 3. Model fit was good for all metabolites with the exception of PA (Table S3). Therefore, the results of PA are not mentioned in Table 3.

**TABLE 3:** Associations between MIND‐NL diet and blood TRP metabolites of the kynurenine and hydroxylation pathways in older adults at risk of cognitive decline.

Path	Crude model Path coefficient (SE), *p*value	Model 1 Path coefficient (SE), *p*value
Tryptophan^a^		
Level‐Level	−0.029 (0.033), *p* = 0.37	−0.001 (0.030), *p* = 0.97
Change‐Change	0.087 (0.107), *p* = 0.42	0.103 (0.120), *p* = 0.39
Kynurenine^a^		
Level‐Level	−0.0720 (0.0427), *p* = 0.09	−0.0350 (0.0386), *p* = 0.37
Change‐Change	−0.0364 (0.0393), *p* = 0.36	−0.0527 (0.0417), *p* = 0.21
Kynurenic acid^a^		
Level‐Level	0.0316 (0.0746), *p* = 0.67	0.0532 (0701), *p* = 0.45
Change‐Change	−0.0483 (0.205), *p* = 0.81	−0.00372 (0.231), *p* = 0.99
Xanthurenic acid^a^		
Level‐Level	−0.00158 (0.0369), *p* = 0.97	0.0182 (0.0336), *p* = 0.59
Change‐Change	0.00699 (0.0881), *p* = 0.94	0.0169 (0.0982), *p* = 86
Quinolinic acid^a^		
Level‐Level	−0.186 (0.0700), ***p* = 0.008**	−0.127 (0.0668), *p* = 0.06
Change‐Change	0.106 (0.162), *p* = 0.51	0.0290 (0.127), *p* = 0.82
Serotonin		
Level‐Level	0.210 (0.131), *p* = 0.11	0.147 (0.127), *p* = 0.25
Change‐Change	−0.0374 (0.171), *p* = 0.83	−0.0500 (0.183), *p* = 0.79
5‐hydroxyindole‐3‐acetic acid^a^		
Level‐Level	−0.122 (0.0659), *p* = 0.06	−0.116 (0.0635), *p* = 0.07
Change‐Change	0.765 (0.554), *p* = 0.17	1.04 (0.687), *p* = 0.13

*Notes*: Level‐level association: Association between latent intercepts. Change‐change association: association between latent difference scores. Model 1: Adjusted for age, sex, baseline and change BMI, baseline and change in physical activity, baseline and change in sleep, baseline and change in perceived stress. Significant p values are indicated in bold.

^a^ Log‐transformed.

Baseline MIND‐NL diet adherence was associated with lower levels of QA (path coefficient −0.186, SE:0.0700, *p* = 0.008), but only within the crude model (level‐level). Significance was lost in the adjusted model, however, a trend remained (path coefficient −0.127, SE:0.0668, *p* = 0.06).

### 3.3 Systemic TRP Metabolite‐Related Ratios of the Kynurenine and Hydroxylation Pathways

Associations between MIND‐NL diet and TRP metabolite‐related ratios are listed in Table 4. Model fit was good for all ratios (Table S3).

**TABLE 4:** Associations between MIND‐NL diet and metabolite‐related ratios of the kynurenine and hydroxylation pathways.

Path	Crude model Path coefficient (SE), *p* value	Model 1 Path coefficient (SE), *p* value
TRP/LNAA ratio		
Level‐Level	−0.0244 (0.0287), *p* = 0.40	−0.00680 (0.0264), *p* = 0.80
Change‐Change	0.0619 (0.0597), *p* = 0.30	0.0567 (0.0691), *p* = 0.41
KYN/LNAA ratio		
Level‐Level	−0.128 (0.0833), *p* = 0.12	−0.0833 (0.0799), *p* = 0.30
Change‐Change	−0.405 (0.196), ***p* = 0.04**	−0.457 (0.206), ***p* = 0.03**
KYN/TRP ratio		
Level‐Level	−0.0688 (0.0871), *p* = 0.43	−0.0559 (0.0864), *p* = 0.52
Change‐Change	−0.525 (0.228), ***p* = 0.02**	−0.558 (0.235), ***p* = 0.02**
5‐HT/TRP ratio		
Level‐Level	0.308 (0.159), *p* = 0.05	0.178 (0.149), *p* = 0.23
Change‐Change	−0.294 (0.291), *p* = 0.31	−0.261 (0.294), *p* = 0.38
KA/QA ratio		
Level‐Level	0.267 (0.104), ***p* = 0.01**	0.201 (0.115), *p* = 0.08
Change‐Change	−0.0418 (0.688), *p* = 0.95	0.419 (0.292), *p* = 0.15

*Note*: Level‐level: Association between latent intercepts. Change‐change: Association between latent difference scores. Model 1: Adjusted for age, sex, baseline and change BMI, baseline and change in physical activity, baseline and change in sleep, baseline and change in perceived stress. LNAAs include: Leucine, isoleucine, tyrosine, phenylalanine, and valine. Significant p values are presented in bold. Abbreviations: KA, kynurenic acid; KYN, kynurenine; LNAA, large neutral amino acids; QA, quinolinic acid; TRP, tryptophan; 5‐HT, serotonin.

Baseline MIND‐NL diet adherence and baseline ratios did not show significant associations (level‐level analyses). On the contrary, changes in MIND‐NL diet adherence were inversely associated with changes in both KYN/LNAA and KYN/TRP ratios (path coefficient −0.457, SE:0.206, *p* = 0.03 and path coefficient −0.558 SE:0.235, *p* = 0.02, respectively).

## 4 Discussion

In the present study, increased adherence to the MIND‐NL diet over a 26‐week intervention period was associated with a reduction in both the kynurenine:large neutral amino acids (KYN/LNAA) ratio and the kynurenine:tryptophan (KYN/TRP) ratio. These findings suggest that greater adherence to the MIND‐NL diet may contribute to decreased activation of the kynurenine pathway.

As far as is known, three independent research groups studied an (adapted) MIND diet in relation to tryptophan metabolites [18, 19, 31]. In all three studies, the focus was on untargeted metabolomics in blood plasma, assessing metabolites of multiple metabolic pathways. Two out of these three studies showed that the MIND diet was cross‐sectionally inversely associated with KYN levels [18, 31]. The other study assessed the effect of a Korean modified MIND diet in older women without dementia, and showed that serotonin (5‐HT) was significantly increased within the intervention group after a 12‐week MIND diet intervention [19]. Our results did not directly confirm the association between MIND diet and KYN at the single metabolite level. However, the reduced KYN/TRP ratio with increased MIND‐NL adherence might reflect an interplay between reduced kynurenine and increased tryptophan levels in our study population, regulated by the activity of indoleamine 2, 3‐dioxygenase (IDO). One key difference between our study and previous research is the sample size. While our study included 82 participants, the two studies showing the MIND‐kynurenine association had considerably larger sample sizes of 807 and 3908 participants. Another difference is the study population: previous studies included a population‐cohort, while our study included a population at risk for cognitive decline based on cardiovascular risk factors.

The KYN/TRP ratio has been inversely associated with cognitive function and AD before, although the evidence remains inconclusive [32, 33, 34]. A systematic review previously showed that studies support cross‐sectional positive associations between adherence to the MIND diet and both global cognition and episodic memory, as well as longitudinal associations with reduced dementia incidence [20]. Taken together, the existing evidence is consistent with the idea that the MIND diet may influence cognition and dementia through kynurenine pathway alterations, as reflected by the KYN/TRP ratio. Although the HELI study assessed cognitive functioning by means of a neurocognitive test battery, the sample size was too small to fit our model and thus to include the association analyses between MIND‐NL diet and cognition. A study duration of 26 weeks was also likely too short to assess changes in cognitive outcomes. Accordingly, we could not corroborate that MIND‐NL diet affects cognition via the tryptophan pathway.

Both the KYN/TRP and KYN/LNAA ratios are indicators of a reduced activation of the kynurenine pathway. A decreased KYN/LNAA ratio in our study population might indicate less transport of KYN over the BBB and, therefore, less KYN available in the brain. KYN/TRP ratio is an indicator of IDO activity. IDO, the rate‐limiting enzyme to transform TRP to KYN, is known to be activated as an inflammatory response [14]. The MIND‐NL diet is considered anti‐inflammatory due to its inclusion of antioxidant‐rich foods (e.g., vitamin C and E, polyphenols, and n‐3 fatty acids), which may contribute to a reduction in IDO activity. IDO1 inhibitors are currently investigated as pharmacotherapy for treating early‐stage AD. Research has shown that in preclinical in‐vitro and mouse models, inhibiting IDO1 restored glycolysis in astrocytes and improved hippocampal glucose metabolism [35]. IDO1 thus becomes involved in two hallmarks of neurodegenerative diseases, inflammation and reduced brain glucose metabolism. Taken together, the KYN/TRP ratio might represent a potential intermediate marker for neurodegenerative diseases.

The level‐level analysis implied that higher MIND‐NL diet adherence was associated with lower plasma levels of QA. QA stimulates NMDA receptors, which can lead to excessive calcium influx into neurons and subsequent cell damage (excitotoxicity). Furthermore, QA is involved in the production of ROS and neuroinflammation. Of all the neurotoxic metabolites of the kynurenine pathway, especially plasma QA was found to be associated with poor cognition in older AD patients [36]. Unfortunately, these MIND‐NL diet and QA associations were not confirmed within the change‐change analysis.

Finally, although the primary focus was on the kynurenine and hydroxylation pathways, plasma levels of gut microbiota‐produced tryptophan metabolites have also been quantified and explored (Table S4). Unexpectedly, no significant associations were noticed.

While interesting leads for future research have been found, several limitations have to be acknowledged. Although we observed good overall model fit indices, the sample size was relatively small given the number of estimated parameters in the latent change models (LCMs) (*k* = 14 crude model, *k* = 34 in adjusted model). In particular, the adjusted model may have been susceptible to limited stability and generalizability of the effect sizes. Nevertheless, the consistency in direction of effect sizes and magnitude of standard errors between the crude and adjusted model suggest findings may reflect meaningful underlying associations, though caution is warranted due to limited power. Additionally, the models could not be further extended due to the limited sample size. As a result, we could not include the intervention group as a separate moderator, while it would have been valuable to explore possible differences in effect size between the two intervention groups. Due to the sample size, correction for multiple testing was not included. Moreover, intake of statins and other cholesterol‐lowering drugs has been associated with lowered kynurenine levels [37]. The association of diet on kynurenine levels might therefore be diminished in the current study. Furthermore, to evaluate how much of the effect size of the association between MIND‐NL diet scores and KYN/TRP ratios may be explained by inflammation status, a mediation analysis would have been insightful. Nevertheless, it should be noted that CRP was measured from finger‐prick blood samples, which are less sensitive to low‐grade inflammation compared to high‐sensitivity CRP (hs‐CRP) assays. For these reasons, future studies with a larger sample size and inclusion of hs‐CRP are recommended.

Additionally, we did not have data of all metabolites generated through the KYN pathway, while it is known that also 3‐hydroxykynurenine and 3‐hydroxyanthranilic acid bear neurotoxic and/or neuroprotective potential [36]. Moreover, blood‐based metabolite assessment may not accurately reflect brain metabolism. However, a previous study showed that plasma concentrations of TRP, KYN, PA, QA, and KYN/TRP ratios positively correlated with their corresponding concentrations in cerebrospinal fluid (CSF) [38].

In the current study, data were only available at baseline and at 26 weeks. Ideally, an intermediate assessment of MIND diet adherence and accompanying TRP metabolite measurements would have been included to better assess MIND diet compliance throughout the study and to strengthen the LCMs. However, most dietary modifications were promoted during the first 11 weeks of the intervention, after which the focus was on maintaining these changes. Therefore, substantial dietary modifications in the final month of the intervention, as captured by the 26‐week FFQ assessment, are not expected compared with a potential intermediate dietary measurement. Additionally, the kynurenine pathway metabolites have been shown to be stable over an extended time period, thereby showing the suitability of the use of an FFQ, assessing habitual intake, compared to food records, which assess more recent dietary changes [39].

Strengths of this study are control for other lifestyle components, namely physical activity, sleep, and perceived stress, impacting the kynurenine and hydroxylation pathways [40, 41]. This is also the first study of its kind, assessing actual changes in diet adherence with changes in plasma TRP metabolite levels of the kynurenine and hydroxylation pathways. In our current analysis, we applied LCMs, a method more commonly used in the field of psychology and neuroscience, but not yet widely applied in nutrition and biomarker research. This methodology also has potential in the field of nutrition, as it can capture within‐person dietary change over time and is more precise than traditional methods by separating true change from residual variation.

To conclude, we demonstrated that the MIND‐NL diet might particularly be associated with related metabolites of the kynurenine pathway. However, future studies with larger sample sizes are needed to confirm these associations. The potential mediating effect of inflammation status is especially of interest for follow‐up investigations, as it may provide important insights into the consecutive mechanisms affected by tryptophan metabolite alterations under the influence of the MIND diet.

## Ethics Statement

The HELI study has been reviewed and accepted according to the Medical Research Involving Human Subjects Act (WMO; “Wet Medisch‐wetenschappelijk Onderzoek met mensen”) by the accredited Medical Research Ethics Committee (MREC) Oost‐Nederland on December 2, 2021 (ToetsingOnline filenumber NL78263.091.21, ClinicalTrials.gov ID NCT05777863).

## Conflicts of Interest

The authors declare no conflicts of interest.

## Supporting information




**Supporting File**: mnfr70377‐sup‐0001‐tablesS1‐S5.docx.

## Data Availability

The data that support the findings of this study are available from the corresponding author upon reasonable request.
