# Supplementary material for: Association Between the Dutch Mediterranean‐Dietary Approaches to Stop Hypertension Intervention for Neurodegenerative Delay (MIND‐NL) Diet Adherence and Systemic Tryptophan Metabolites in Older Adults at Risk of Cognitive Decline: An Exploratory Study
Source: Mol Nutr Food Res. 2026 Jan 16;70(1):e70377. doi: 10.1002/mnfr.70377 (PMC12810439; doi:10.1002/mnfr.70377)
Supplement: Supplementary file 1 — Supporting File: mnfr70377‐sup‐0001‐tablesS1‐S5.docx. [file MNFR-70-e70377-s001.docx]

**Supplementary Table 1.** Modifiable cardiovascular risk factor scale

| Risk Factor | Point(s) |
| --- | --- |
| BMI ≥ 25kg/m^2^ (overweight) | 1 |
| Physical inactivity (below the 2020 WHO guidelines: <300 minutes of moderate intensity aerobic physical activity or <150 minutes of vigorous intensity aerobic physical activity per week, spread out over several days | 1 |
| Hypertension (systolic blood pressure ≥140 mmHg, and diastolic blood pressure ≥90 mmHg | 1  (2 points are assigned if hypertension is not being actively treated with antihypertensive medication, given the increased cardiovascular burden) |
| Hypercholesterolemia (total cholesterol >5 mmol/L, or LDL-cholesterol >3 mmol/L) | 1 |
| Diabetes type-II | 1 |
| Mild cardiovascular disease (e.g. intermittent claudication, varicose veins; In contrast, moderate or severe cardiovascular disease such as stroke, angina pectoris, heart failure, myocardial infarction or revascularization surgery in the last 12 months before pre-screening are exclusion criteria. | 1 |

**Supplementary Table 2.** Components of the Dutch versions of the Mediterranean-DASH Diet Intervention for Neurodegenerative Delay (MIND-NL) diet and their cut-off values.

| MIND-NL food groups | Score | | |
| --- | --- | --- | --- |
|  | **0** | **0.5** | **1** |
| Green leafy vegetables | ≤29 g/day | >29 - <100 g/day | ≥100 g/day |
| Other vegetables | <71 g/day | ≥ 71 -<100 g/day | ≥100 g/day |
| Berries and strawberries | <7 g/day | ≥7 - <36 g/day | ≥36 g/day |
| Legumes | <9 g/day | ≥9 - <26 g/day | ≥26 g/day |
| Nuts | <3 g/day | ≥3 - <20 g/day | ≥20 g/day |
| Fish (not fried) | <13 g/day | 13 g/day | >13 g/day |
| Whole grains | <30 g/day | ≥30 - <90 g/day | ≥90 g/day |
| Poultry | <14 g/day | ≥14 - <29 g/day | ≥29 g/day |
| Olive oil | <15 g/day | ≥15 - <30 g/day | ≥30 g/day |
| Butter and stick margarine | ≥10 g/day | >5 - <10 g/day | ≤5 g/day |
| Full-fat cheese | ≥30 g/day | >9 - <30 g/day | ≤9 g/day |
| Red and processed meat | ≥100 g/day | >43 - <100 g/day | ≤43 g/day |
|  |  |  |  |
| Wine | >100 ml/d | NA | ≤100 ml/d |
| Take out, fried foods and snacks | >3 serving eq/wk | >1 - ≤3 serving eq/wk | ≤1 serving eq/wk |
| Cookies, pastries and sweets | >4 serving eq/wk | >2 - ≤4 serving eq/wk | ≤2 serving eq/wk |

**Supplementary Table 3.** Model fit indices of bivariate Latent Change Models (LCMs)

|  | Chi2 (p-value) | DF | CFI | RMSEA | SRMR |
| --- | --- | --- | --- | --- | --- |
| *Single metabolites* |  |  |  |  |  |
| TRP^1)^ |  |  |  |  |  |
| Crude model | 0.42 (0.94) | 3 | 1.00 | 0.00 | 0.02 |
| Model 1 | 19.80 (0.65) | 23 | 1.00 | 0.00 | 0.04 |
| KYN^1)^ |  |  |  |  |  |
| Crude model | 0.561 (0.91) | 3 | 1.00 | 0.00 | 0.02 |
| Model 1 | 19.174 (0.69) | 23 | 1.00 | 0.02 | 0.04 |
| KYNA^1)^ |  |  |  |  |  |
| Crude model | 0.815 (0.85) | 3 | 1.00 | 0.00 | 0.03 |
| Model 1 | 17.259 (0.80) | 23 | 1.00 | 0.00 | 0.04 |
| XA^1)^ |  |  |  |  |  |
| Crude model | 1.846 (0.61) | 3 | 1.00 | 0.00 | 0.05 |
| Model 1 | 19.607 (0.67) | 23 | 1.00 | 0.00 | 0.04 |
| QA^1)^ |  |  |  |  |  |
| Crude model | 1.666 (0.65) | 3 | 1.00 | 0.00 | 0.03 |
| Model 1 | 18.177 (0.75) | 23 | 1.00 | 0.00 | 0.04 |
| PA^1)^ |  |  |  |  |  |
| Crude model | 12.315 (0.006) | 3 | 0.80 | 0.20 | 0.09 |
| Model 1 | 35.391 (0.048) | 23 | 0.80 | 0.09 | 0.05 |
| 5-HT |  |  |  |  |  |
| Crude model | 4.836 (0.18) | 3 | 0.96 | 0.09 | 0.06 |
| Model 1 | 23.845 (0.41) | 23 | 0.98 | 0.02 | 0.04 |
| 5-HIAA^1)^ |  |  |  |  |  |
| Crude model | 3.710 (0.30) | 3 | 0.97 | 0.05 | 0.07 |
| Model 1 | 16.726 (0.82) | 23 | 1.00 | 0.00 | 0.04 |
|  |  |  |  |  |  |
| *Ratios* |  |  |  |  |  |
| TRP/LNAA |  |  |  |  |  |
| Crude model | 0.310 (0.96) | 3 | 1.00 | 0.00 | 0.02 |
| Model 1 | 21.942 (0.52) | 23 | 1.00 | 0.00 | 0.04 |
| KYN/LNAA |  |  |  |  |  |
| Crude model | 3.207 (0.36) | 3 | 1.00 | 0.03 | 0.04 |
| Model 1 | 25.162 (0.34) | 23 | 0.97 | 0.04 | 0.05 |
| KYN/TRP |  |  |  |  |  |
| Crude model | 1.207 (0.75) | 3 | 1.00 | 0.00 | 0.03 |
| Model 1 | 27.488 (0.24) | 23 | 0.93 | 0.05 | 0.05 |
| 5-HT/TRP |  |  |  |  |  |
| Crude model | 4.032 (0.26) | 3 | 0.98 | 0.06 | 0.05 |
| Model 1 | 22.083 (0.52) | 23 | 1.00 | 0.00 | 0.04 |
| KA/QA |  |  |  |  |  |
| Crude model | 0.171 (0.98) | 3 | 1.00 | 0.00 | 0.01 |
| Model 1 | 10.4 (0.99) | 23 | 1.00 | 0.00 | 0.03 |

KYNA: Kynureninc Acid, KYN: Kynurenine, QA: Quinolinic acid, TRP: Tryptophan, XA: Xanthurenic acid, 5-HT: Serotonin, 5-HIAA: 5-hydroxyindole-3-acetic acid
^1)^ log transformed variable

**Supplementary Table 4.** Associations between MIND-NL diet with gut microbiota related tryptophan metabolites.

| Path | Crude model  Path coefficient (SE), p-value | Model 1 Path coefficient (SE), p-value |
| --- | --- | --- |
| Indole-3-lactic acid (ILA) ^1)^ |  |  |
| Level-Level | 0.0290 (0.0702), p=0.68 | 0.0758 (0.0632), p=0.23 |
| Change-Change | -0.0210 (0.0227), p=0.36 | -0.0269 (0.0229), p=0.24 |
| Indole-3-propionic acid (IPA) ^1)^ |  |  |
| Level-Level | 0.0847 (0.111), p=0.45 | 0.0933 (0.107), p=0.38 |
| Change-Change | 0.0489 (0.0707), p=0.49 | 0.0703 (0.0714), p=0.32 |
| Indole-3-acriloylglycine (IAG) ^1)^ |  |  |
| Level-Level | -0.194 (0.130), p=0.14 | -0.115 (0.127), p=0.36 |
| Change-Change | 0.0902 (0.326), p=0.78 | -0.114 (0.346), p=0.74 |
| Indoxyl sulfate (IS) ^1)^ |  |  |
| Level-Level | -0.167 (0.117), p=0.15 | -0.166 (0.117), p=0.16 |
| Change-Change | -0.151 (0.151), p=0.32 | -0.0214 (0.142), p=0.88 |
| Indole-3-acetic acid (IAA) ^1)^ |  |  |
| Level-Level | 0.0773 (0.0850), p=0.36 | 0.0371 (0.0875), p=0.67 |
| Change-Change | -0.0401 (0.142), p=0.78 | 0.0925 (0.115), p=0.42 |
| Indole-3-acetic acid methyl ester (IAA ME) ^2)^ |  |  |
| Level-Level | -0.233 (0.187), p=0.21 | -0.142 (0.168), p=0.40 |
| Change-Change | 0.0172 (0.0791), p=0.83 | 0.0292 (0.0841), p=0.73 |
| Indole-3-carboxaldehyde (ICARB) ^2)^ |  |  |
| Level-Level | -0.0380 (0.173), p=0.83 | 0.0250 (0.163), p=0.88 |
| Change-Change | 0.0151 (0.0682), p=0.83 | -0.0147 (0.0719), p=0.84 |

^1)^ log transformed variable, ^2)^ scaled variable

**Supplementary Table 5.** Model fit indices of bivariate Latent Change Models (LCMs) of gut microbiota related tryptophan metabolites

|  | Chi2 (p-value) | DF | CFI | RMSEA | SRMR |
| --- | --- | --- | --- | --- | --- |
| Indole-3-lactic acid (ILA) ^1)^ |  |  |  |  |  |
| Crude model | 0.275 (0.97) | 3 | 1.00 | 0.00 | 0.02 |
| Model 1 | 9.752 (0.99) | 23 | 1.00 | 0.00 | 0.03 |
| IPA ^1)^ |  |  |  |  |  |
| Crude model | 5.773 (0.12) | 3 | 0.96 | 0.11 | 0.06 |
| Model 1 | 25.336 (0.33) | 23 | 0.97 | 0.04 | 0.04 |
| Indole-3-acriloylglycine ^1)^ |  |  |  |  |  |
| Crude model | 3.742 (0.29) | 3 | 0.98 | 0.05 | 0.05 |
| Model 1 | 24.242 (0.39) | 23 | 0.98 | 0.03 | 0.05 |
| Indoxyl sulfate (IS) ^1)^ |  |  |  |  |  |
| Crude model | 0.973 (0.81) | 3 | 1.00 | 0.00 | 0.03 |
| Model 1 | 21.359 (0.56) | 23 | 1.00 | 0.00 | 0.04 |
| Indole-3-acetic acid (IAA) ^1)^ |  |  |  |  |  |
| Crude model | 2.442 (0.49) | 3 | 1.00 | 0.00 | 0.04 |
| Model 1 | 15.556 (0.87) | 23 | 1.00 | 0.00 | 0.04 |
| Indole-3-acetic acid methyl ester (IAA ME) ^2)^ |  |  |  |  |  |
| Crude model | 6.683 (0.08) | 3 | 0.90 | 0.12 | 0.07 |
| Model 1 | 27.334 (0.24) | 23 | 0.92 | 0.05 | 0.05 |
| Indole-3-carboxaldehyde (ICARB) ^2)^ |  |  |  |  |  |
| Crude model | 1.974 (0.58) | 3 | 1.00 | 0.00 | 0.04 |
| Model 1 | 23.239 (0.45) | 23 | 1.00 | 0.01 | 0.04 |

^1)^ log transformed variable, ^2)^ scaled variable
